# Supplementary material for: Bioinspired soft robots based on organic polymer-crystal hybrid materials with response to temperature and humidity
Source: Nat Commun. 2023 Apr 21;14:2287. doi: 10.1038/s41467-023-37964-1 (PMC10121608; doi:10.1038/s41467-023-37964-1)
Supplement: Supplementary file 1 — Supplementary Information [file 41467_2023_37964_MOESM1_ESM.pdf]

Supplementary Information

Bioinspired Soft Robots based on Organic Polymer-Crystal Hybrid Materials with Response to Temperature and Humidity

Xuesong Yang[1], Linfeng Lan[1], Xiuhong Pan[1], Qi Di[1], Xiaokong Liu[1], Liang Li[2,3]*, Panče Naumov[2,4,5]*, and Hongyu Zhang[1]*

[1]State Key Laboratory of Supramolecular Structure and Materials, College of Chemistry, Jilin University, Changchun 130012, P. R. China

[2]Smart Materials Lab, New York University Abu Dhabi, PO Box 129188, Abu Dhabi, UAE

[3]Department of Sciences and Engineering, Sorbonne University Abu Dhabi, PO Box 38044, Abu Dhabi, UAE

[4]Research Center for Environment and Materials, Macedonian Academy of Sciences and Arts, Bul. Krste Misirkov 2, MK–1000 Skopje, Macedonia

[5]Molecular Design Institute, Department of Chemistry, New York University, 100 Washington Square East, New York, NY 10003, USA

*Corresponding authors. Emails for correspondence: liang.li@sorbonne.ae (L. L.); pance.naumov@nyu.edu (P. N.); hongyuzhang@jlu.edu.cn (H. Z.)

Supplementary Methods

All solvents and starting materials for syntheses were purchased from commercial sources and were used as received without further purification. Poly(diallyldimethylammonium chloride) (PDDA, Mw. 200,000–350,000) (99%), poly(sodium styrene sulfonate) (PSS, Mw. 70,000) (99%), polyvinyl alcohol (PVA, mw. 105,000) (99%), and glutaraldehyde (GA) (98%) were purchased from Energy Chemical. PDDA and PSS aqueous solutions were at a concentration of 1.0 mg/mL. To prepare the 5% PVA aqueous solution, after mixing PVA granules with pure water in mass ratio PVA : H_2O = 5 : 95, the suspension was first stirred mechanically at room temperature for 2 h, and then stirred at 95 °C in a water bath for 2 h. A clear 5% PVA aqueous solution was obtained. The ^1H and $^{13}C\{^1H\}$ NMR spectra were recorded on 500 MHz spectrometers with tetramethylsilane as the internal standard. The mass spectra were recorded on a Thermo Fisher ITQ1100 mass spectrometer. The elemental analyses were performed on an Elementar Vario Micro Cube analyzer. The scanning electron microscopy (SEM) images were obtained on the FEI Quanta 450 operated at 5–10 kV. The infrared spectrum was recorded on a VERTEX 80V infrared spectrometer.

Supplementary Figures

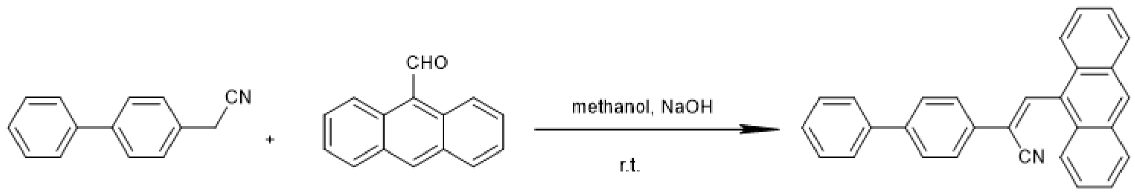

Supplementary Figure 1. The synthetic procedure for compound 1. 4-Biphenylacetonitrile (1.93, 10 mmol) and 9-anthraldehyde (2.06 g, 10 mmol) were added to ethanol (50 mL). NaOH (0.40 g, 10 mmol) was then added, and the mixture was stirred for 4 h at room temperature. The mixture was filtered to have a yellow solid, which was dissolved in dichloromethane and washed with brine. After drying over Na_2SO_4, the solvent was removed by vacuum roto-evaporation. The resulting crude product was purified by column chromatography using dichloromethane as an eluent to obtain compound 1 (3.01 g, 75%) as a yellow powder. ^{1}H NMR (Chloroform-*d*, 500 MHz) δ 8.54 (1H, s), 8.47 (1H, s), 8.10 – 8.00 (4H, m), 7.96 (2H, d, J = 8.0 Hz), 7.77 (2H, d, J = 8.0 Hz), 7.71 – 7.65 (2H, m), 7.58 – 7.44 (6H, m), 7.44 – 7.38 (1H, m) ^{13}C NMR (Chloroform-*d*, 126 MHz) δ 142.73, 139.97, 139.80, 132.18, 131.32, 129.50, 129.14, 129.03, 128.02, 127.90 (2C), 127.15, 126.70, 126.60, 125.56(2C), 125.07, 120.91, 116.53. MS (ESI+): m/z calcd for $C_{29}H_{19}N$ $[M+H]^+$: 381.15; Found: 381.27. Anal. calcd (%) for $C_{29}H_{19}N$: C, 91.31; H, 5.02; N, 3.67. Found: C, 91.37; H, 5.06; N, 3.63.

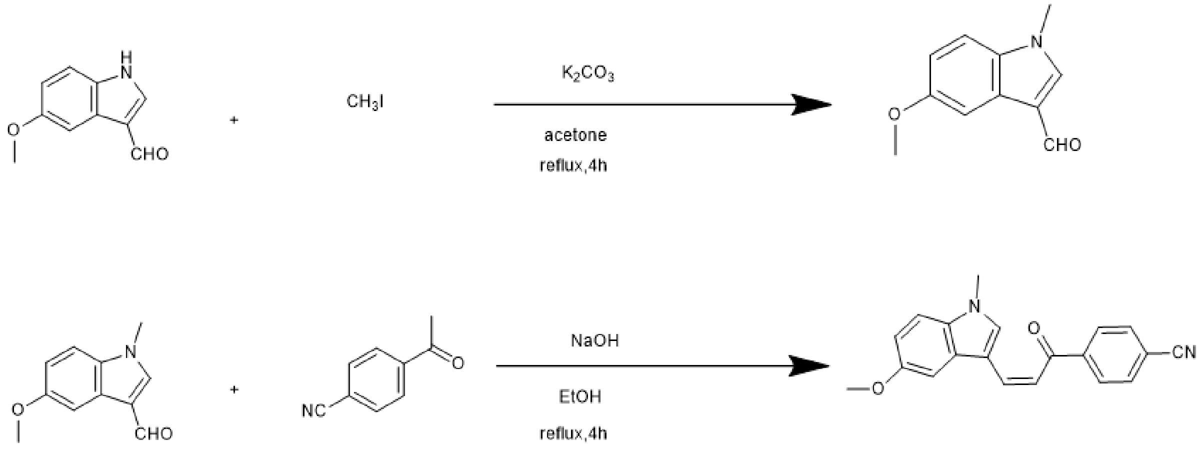

Supplementary Figure 2. The synthetic procedure for compound 2. 5-Methoxy-1*H*-indole-3-carbaldehyde (1.75 g, 10 mmol) was dissolved in 50 mL acetone, and K_2CO_3 (4.15 g, 30 mmol) was added. Then 1 mL CH_3I was injected into the solvent, and the mixture was refluxed for 4 h. After cooling to room temperature, the K_2CO_3 was filtered out, and the solvent was removed by distillation under reduced pressure. 80 mL ethanol, 4-acetylbenzonitrile (1.45 g, 10 mmol) and NaOH (0.08 g, 2 mmol) were added to the residue, and the mixture was refluxed for 4 h. The obtained precipitate was filtered and washed with ethanol to give the crude product, which was purified by vacuum sublimation to produce compound **2** as a yellow solid (1.81 g, 57% yield). ^{1}H NMR (DMSO-d_6, 500 MHz) δ 8.24 (1H, d, J = 1.7 Hz), 8.22 (1H, d, J = 2.0

Hz), 8.13 (1H, s), 8.04 (2H, d, J = 3.7 Hz), 8.02 (1H, d, J = 1.8 Hz), 7.56 (1H, s), 7.50 (1H, d, J = 2.4 Hz), 7.47 (1H, s), 6.97 (1H, d, J = 2.4 Hz), 3.88 (3H, s), 3.84 (3H, s). ^{1}H NMR (DMSO-d_6, 500 MHz) δ 8.22 (2H, d, J = 8.1 Hz), 8.11 (1H, s), 8.06 (1H, d, J = 15.3 Hz), 8.02 (2H, d, J = 8.0 Hz), 7.53 (1H, d, J = 15.4 Hz), 7.50 (1H, d, J = 2.4 Hz), 7.47 (1H, d, J = 8.9 Hz), 6.96 (1H, dd, J = 8.9, 2.3 Hz), 3.88 (3H, s), 3.84 (3H, s). ^{13}C NMR (DMSO-d_6, 126 MHz) δ 188.20, 155.90, 142.63, 140.20, 137.45, 133.54, 133.15(2C), 129.14(2C), 127.03, 118.87, 114.86, 114.67, 112.64, 112.20, 112.05, 103.44, 56.13, 33.76. MS (ESI+): m/z calcd for $C_{20}H_{16}N_2O_2$ $[M+H]^+$: 316.12; Found: 316.18. Anal. calcd (%) for $C_{20}H_{16}N_2O_2$: C, 75.93; H, 5.10; N, 8.86. Found: C, 76.14; H, 5.16; N, 8.77.

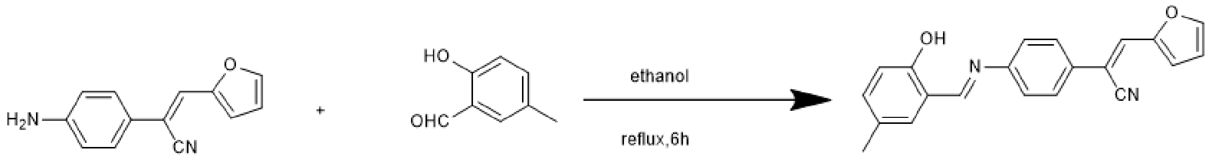

Supplementary Figure 3. The synthetic procedure of compound 3. 2-(4-aminophenyl)-3-(furan-2-yl)acrylonitrile (2.10 g, 10 mmol) and 2-hydroxy-5-methylbenzaldehyde (1.36 g, 10 mmol) were dissolved in ethanol (50 mL). After refluxing for 6 h, the resultant mixture was cooled down to room temperature, and was filtered and washed with ethanol. The crude product was purified by column chromatography using dichloromethane and petroleum ether (V/V= 4:1) as the eluent to produce compound 3 as an orange-red solid (3.02 g, 92.1% yield). ^{1}H NMR (DMSO-d_6, 500 MHz) δ 12.64 (1H, s), 8.97 (1H, s), 8.04 (1H, d, J = 1.8 Hz), 7.95 (1H, s), 7.82 (2H, d, J = 8.5 Hz), 7.54 (2H, d, J = 8.4 Hz), 7.48 (1H, d, J = 2.3 Hz), 7.26 (1H, dd, J = 8.4, 2.2 Hz), 7.18 (1H, d, J = 3.5 Hz), 6.89 (1H, d, J = 8.3 Hz), 6.79 (1H, dd, J = 3.5, 1.8 Hz), 2.29 (3H, s). ^{13}C NMR (DMSO-d_6, 126 MHz) δ 164.08, 158.64, 150.22, 149.16, 146.85, 134.82, 132.74, 132.18, 128.54, 128.28, 126.99, 122.70, 119.52, 118.03, 117.61, 116.99, 113.71, 105.60, 20.41. MS (ESI+): m/z calcd for $C_{21}H_{16}N_2O_2$ $[M+H]^+$: 328.12; Found: 328.05. Anal. calcd (%) for $C_{21}H_{16}N_2O_2$: C, 76.81; H, 4.91; N, 8.53. Found: C, 76.92; H, 4.95; N, 8.63.

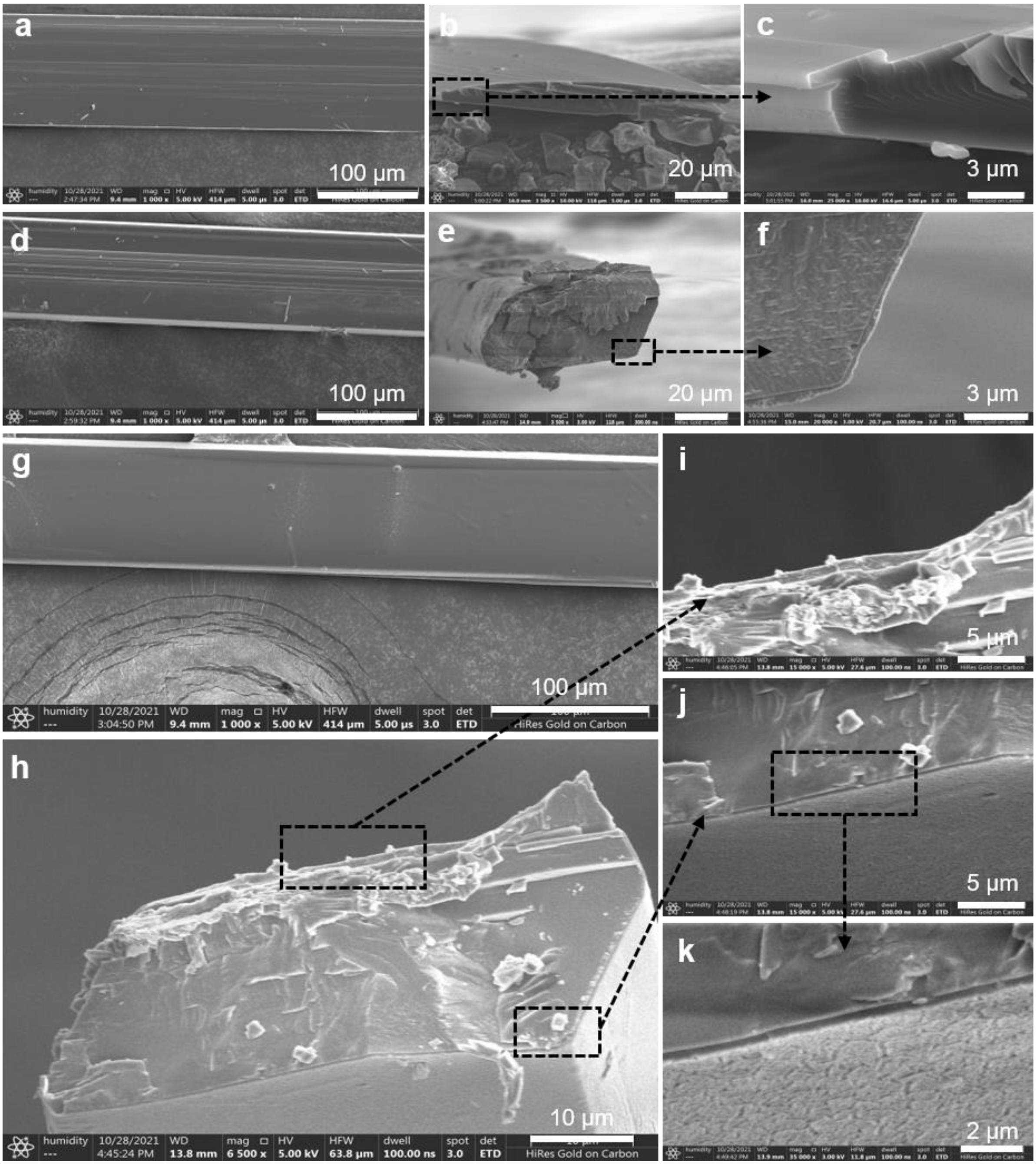

Supplementary Figure 4. Photographs of crystals taken under scanning electron microscope. (a,d,g) Surface of crystal 3 (a), PDDA/PSS//3 (d), and P^2//3 (g). (b,c) Cross-section of crystal 3. (e,f) Cross-section of PDDA/PSS//3. (h–k) Cross-section of P^2//3.

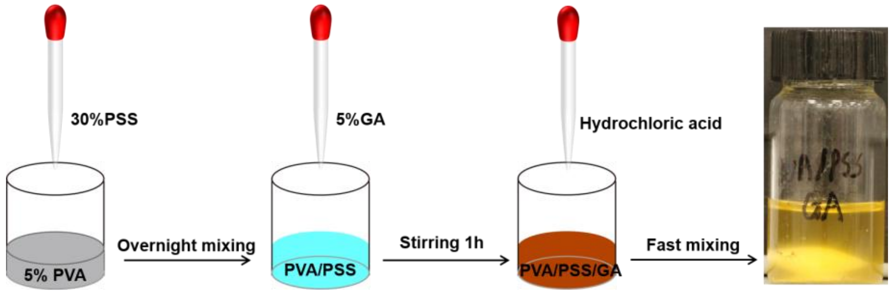

Supplementary Figure 5. Preparation of polymeric materials. Preparation of polyvinyl alcohol/poly(sodium 4-styrenesulfonate)/glutaraldehyde (PVA/PSS/GA). After mixing 5% PVA solution with 30% PSS solution according to the mass ratio (PSS : PVA = 1 : 4) and stirring magnetically overnight, 5% GA solution was added dropwise to the PVA/PSS mixture according to the mass ratio (GA/PVA = 2%) and stirred magnetically for 1 h, and then 2 mL of 2 mol/L hydrochloric acids was added dropwise to the PVA/PSS/GA mixture with rapid stirring, and the polymer material was cross-linked at room temperature.

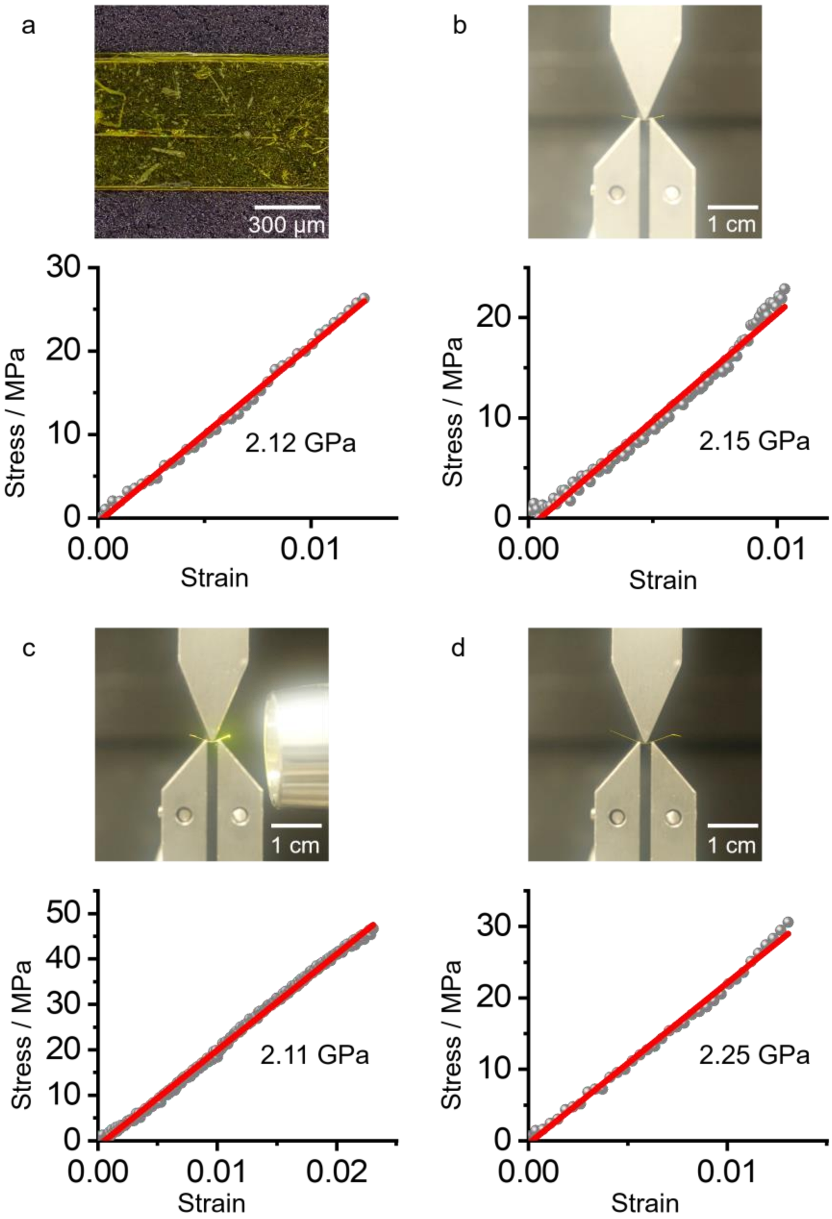

Supplementary Figure 6. Stress-strain profiles of the hybrid materials under different conditions. (a) Crystal 1 at ambient condition. (b) P^2//1 at ambient condition. (c) P^2//1 under UV light irradiation. (d) P^2//1 tested in a stream of air at high humidity.

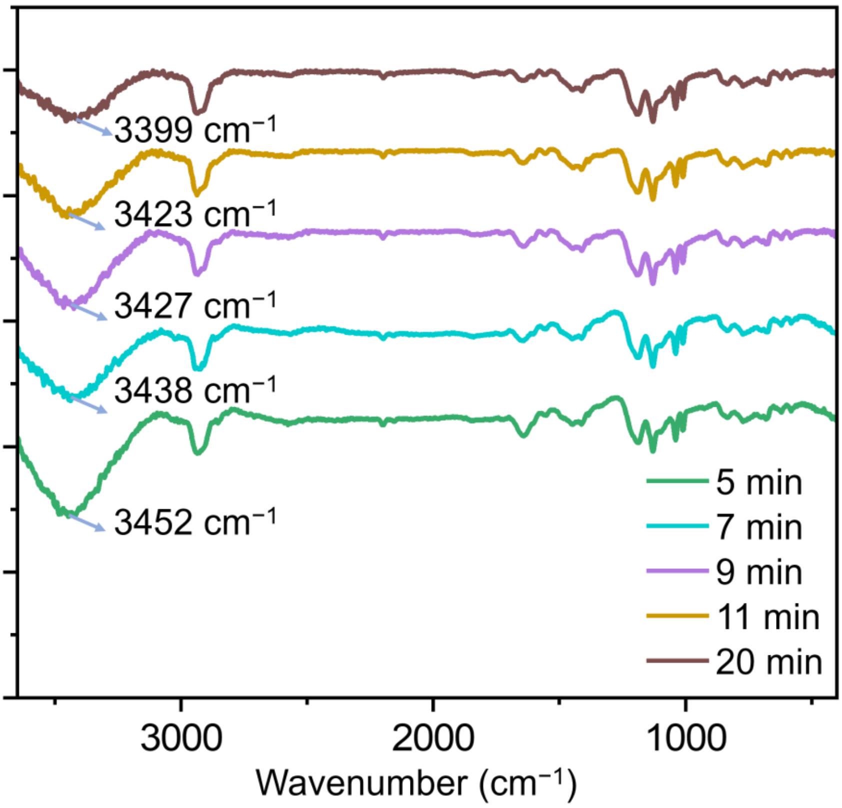

Supplementary Figure 7. Infrared spectra of polymers heated over different times with infrared lamp at 250 W (5 min, 7 min, 9 min, 11 min, and 20 min). The broad, complex band that is attributed to the O–H stretching shifts from (approximately) 3452 cm^{-1} to 3399 cm^{-1} with increasing heating time, qualitatively indicating changes in the hydrogen-bonded network.

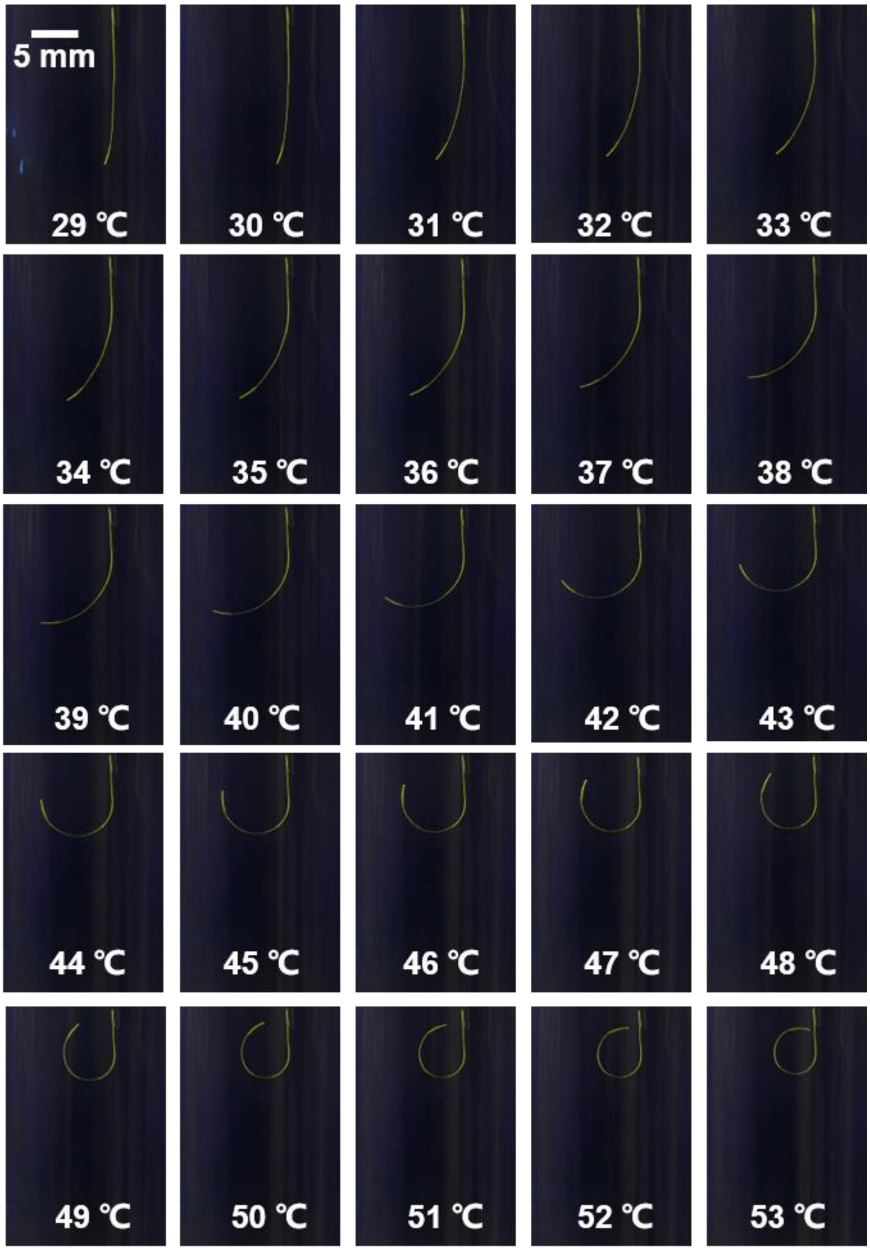

Supplementary Figure 8. Temperature response of hybridized materials. The photographs of P^2//1 bending at different temperature.

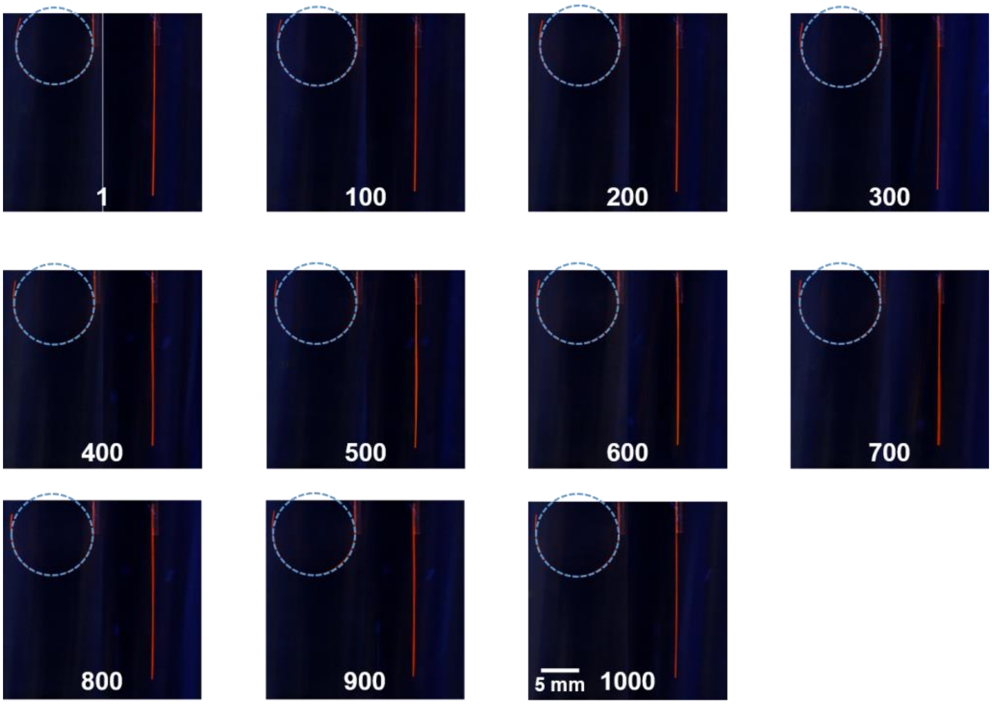

Supplementary Figure 9. Durability of hybrid materials. Optical images of bending durability tests perforfmed at humidities of 85.1% and 39.6% for P^2//3.

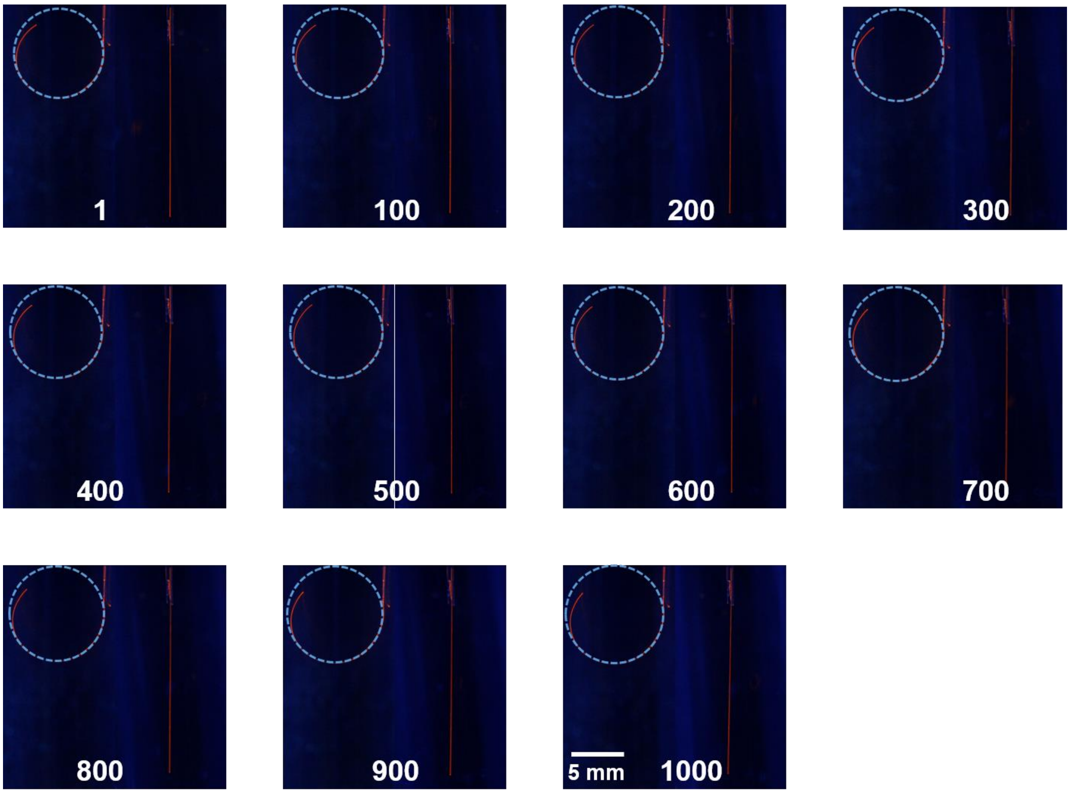

Supplementary Figure 10. Durability of hybrid materials. Optical images of bending durability tests performed between 20 °C and 45 °C for P^2//3.

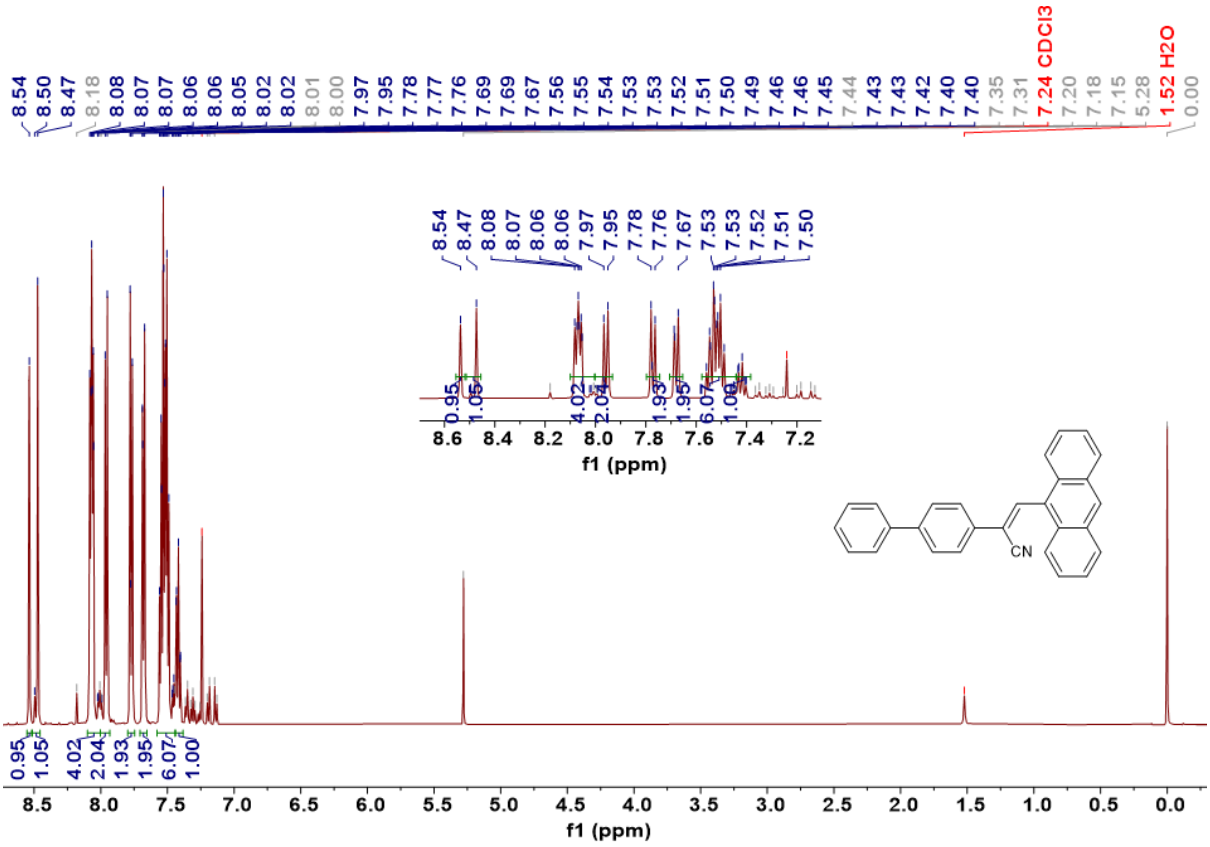

Supplementary Figure 11. ^{1}H NMR spectrum of compound 1 (chloroform-*d*, 500 MHz).

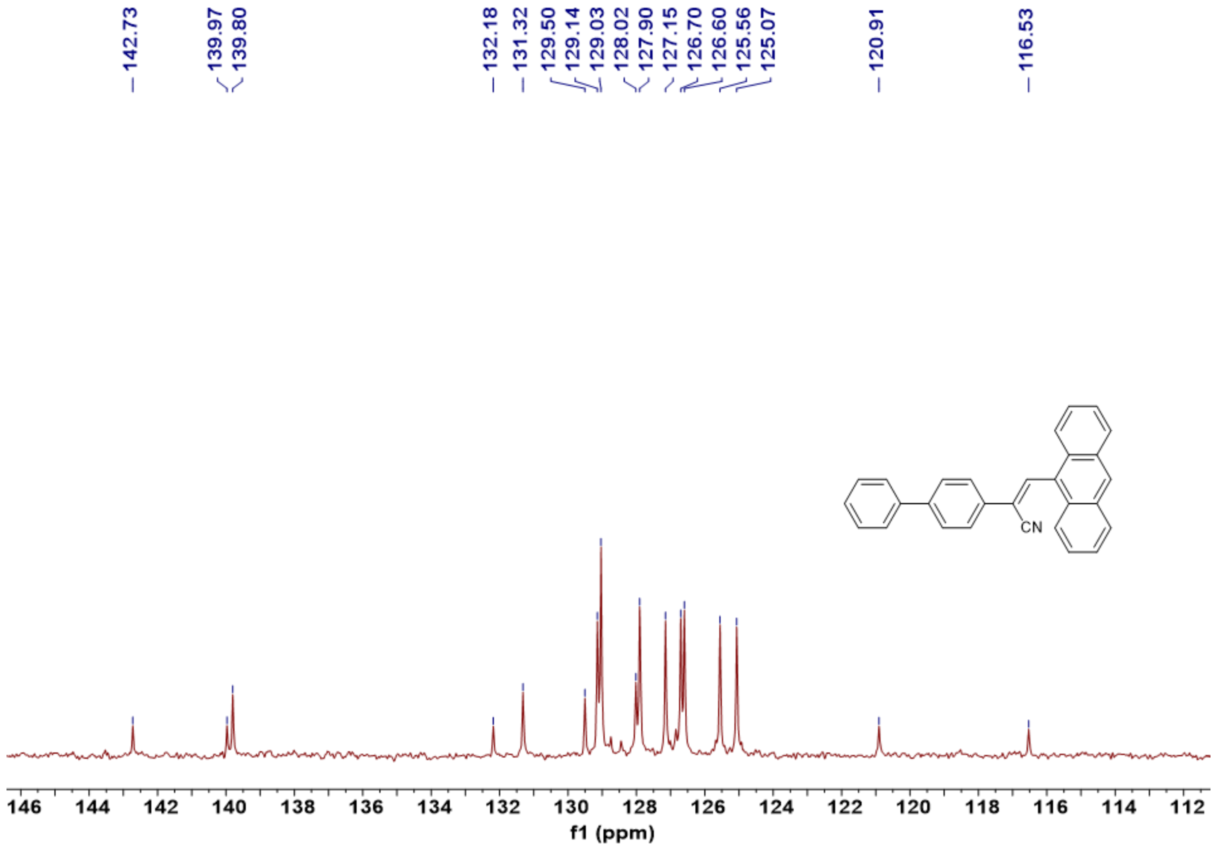

Supplementary Figure 12. $^{13}C\{1H\}$ NMR spectrum of compound 1 (chloroform-*d*, 126 MHz).

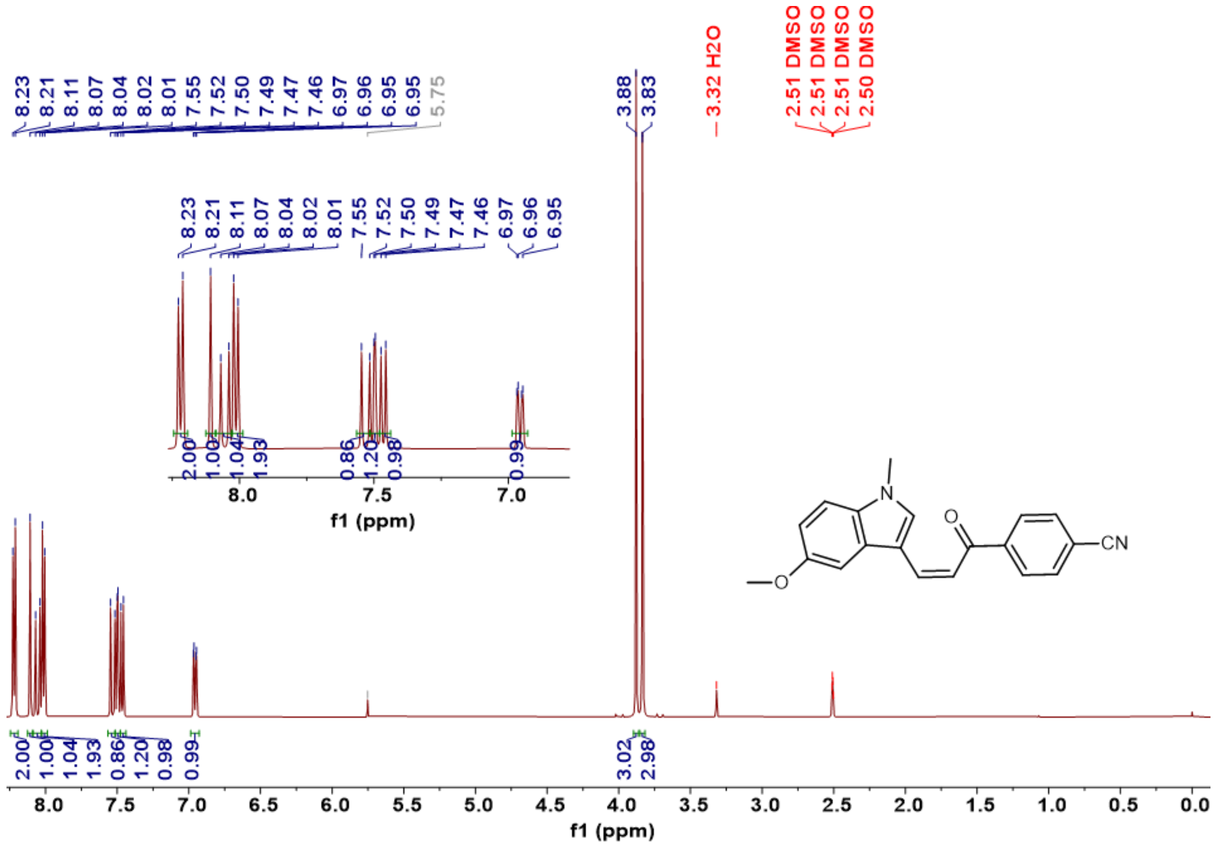

Supplementary Figure 13. ^{1}H NMR spectrum of compound 2 (DMSO-d_6, 500 MHz).

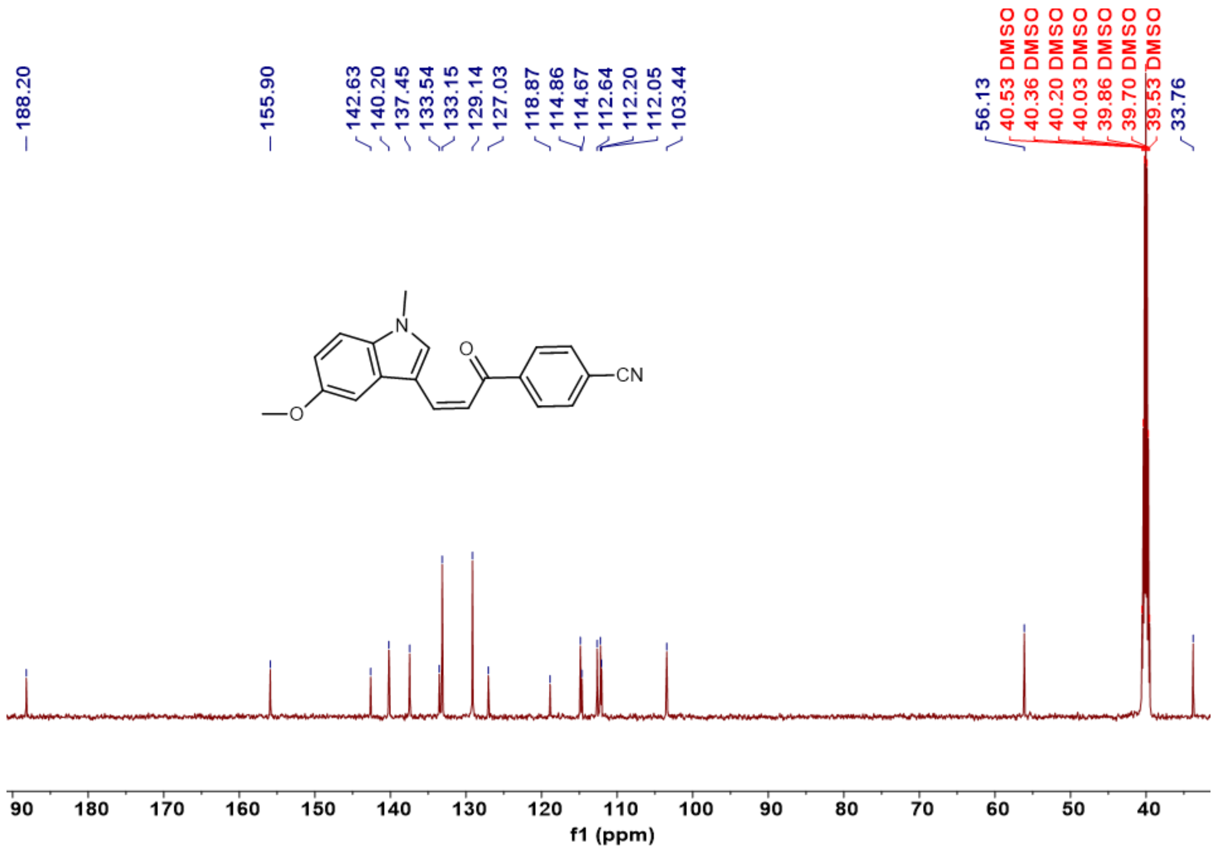

Supplementary Figure 14. ^{13}C{1H} NMR spectrum of compound 2 (DMSO-d_6, 126 MHz).

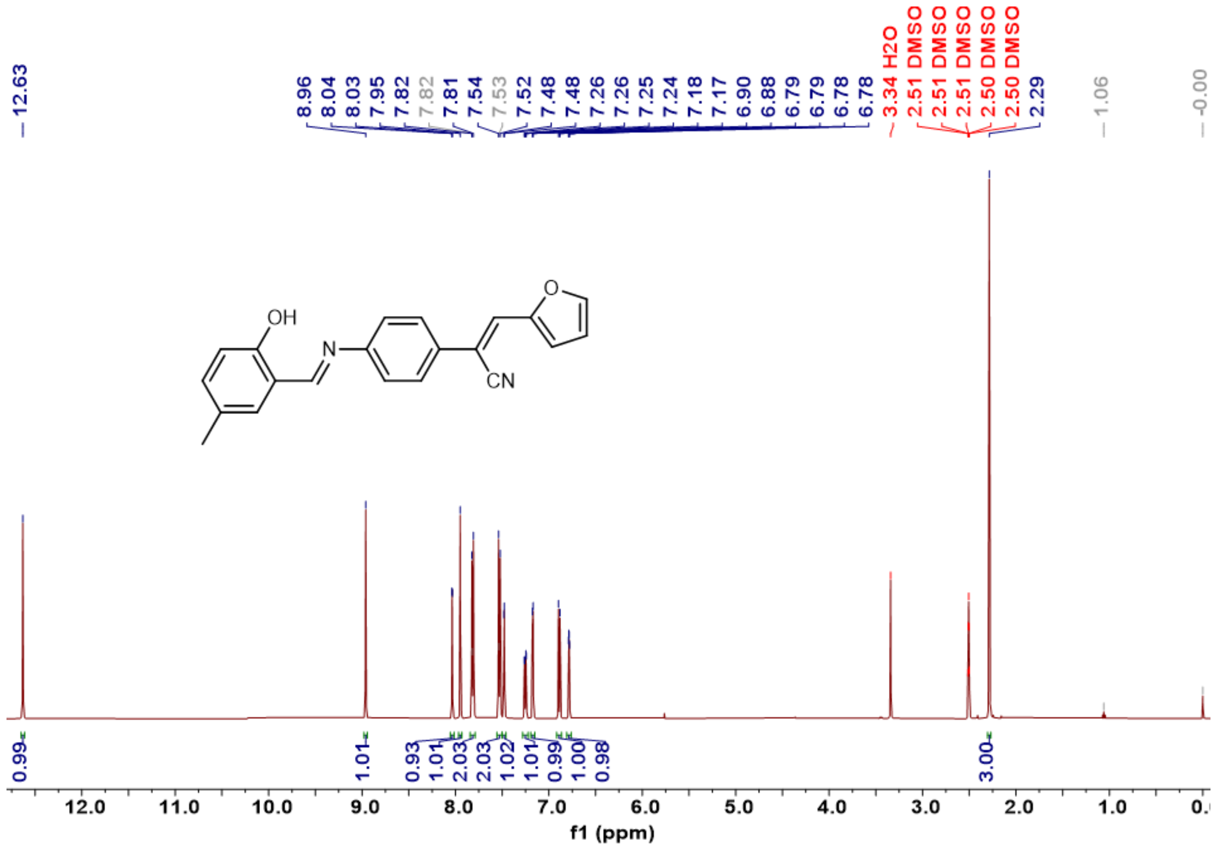

Supplementary Figure 15.^{1}H NMR spectrum of compound 3 (DMSO-d_6, 500 MHz).

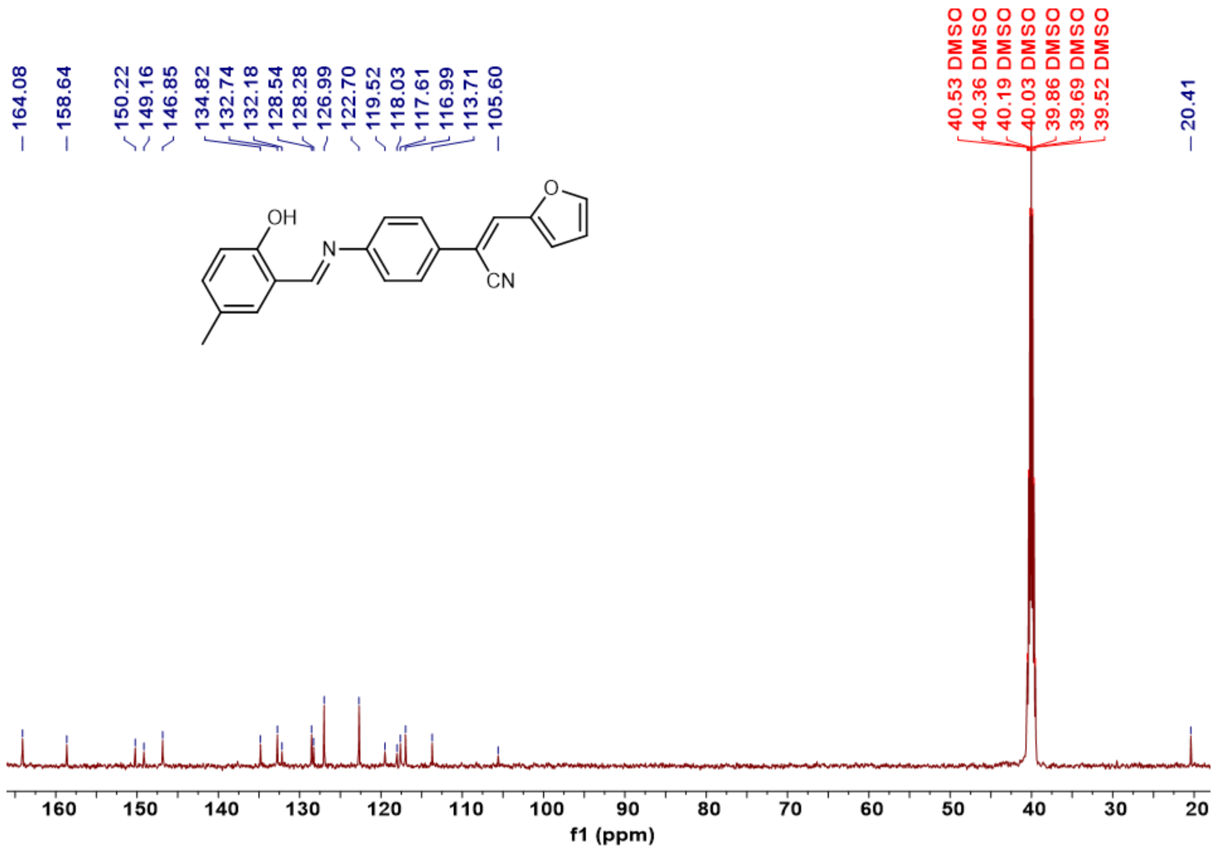

Supplementary Figure 16. $^{13}C\{1H\}$ NMR spectrum of compound 3 (DMSO-d_6, 126 MHz).

Supplementary Tables

Supplementary Table 1. Length, width, thickness of crystals of compounds 1–3

Compound	1	2	3
Length	1–3 cm	2–4 cm	1–3 cm
Width	80–300 μm	108–219 μm	160–220 μm
Thickness	20–130 μm	40–112 μm	10–22 μm
