# Supplementary material for: Bioinspired soft robots based on organic polymer-crystal hybrid materials with response to temperature and humidity
Source: Nat Commun. 2023 Apr 21;14:2287. doi: 10.1038/s41467-023-37964-1 (PMC10121608; doi:10.1038/s41467-023-37964-1)
Supplement: Supplementary file 3 — Description of Additional Supplementary Files [file 41467_2023_37964_MOESM3_ESM.pdf]

Legends to the Supplementary Movies

Supplementary Movie 1. Bending of the organic polymer-crystal hybrid materials induced by humidity.

Supplementary Movie 2. Bending of the organic polymer-crystal hybrid materials induced by temperature.

Supplementary Movie 3. Simulation of a plant tendril by using hybrid crystal-polymer material.

Supplementary Movie 4. Simulation of a spider-like motion by using organic polymer-crystal hybrid materials.

Supplementary Movie 5. Gripping device based on organic polymer-crystal hybrid materials.

Supplementary Movie 6. “Walking“ of the organic polymer-crystal hybrid materials across a surface.
